# Incidence of neurological complications following pediatric heart surgery and its association with neutrophil‐to‐lymphocyte ratio

**DOI:** 10.1002/hsr2.1077

**Published:** 2023-01-18

**Authors:** Alireza Abdshah, Mohammadreza Mirzaaghayan, Morteza Heidari, Sara Memarian, Mobina Amanollahi, Azadeh Nazeri, Behdad Gharib

**Affiliations:** ^1^ School of Medicine Tehran University of Medical Sciences Tehran Iran; ^2^ Department of Public Health Sciences, Division of Biostatistics University of Miami Miller School of Medicine Miami Florida USA; ^3^ Children's Medical Center Tehran University of Medical Sciences Tehran Iran

**Keywords:** neurological complications, NLR, RACHS, surgical risk

## Abstract

**Background and Aims:**

Due to increased rate of open‐heart surgeries in children, postsurgical mortality and morbidities have increasingly gained attention. Neurological complications are some of the most important postsurgical events. However, the number of studies regarding postsurgical neurological complications seems to be inadequate. We aimed to study the incidence of neurological complications following cardiac surgery in the pediatric cardiac intensive care unit (ICU) of the children's medical center.

**Methods:**

This cross‐sectional study was conducted from March to September 2019. We included all of the children who underwent cardiac surgery and were admitted to ICU at CMC. We collected their demographic data, lab test results (white blood cell count, absolute neutrophile and lymphocyte counts) and calculated their Risk Adjustment for Congenital Heart Surgery (RACHS) score. We then documented neurological adverse events and investigated the associations between those events and the patients' data.

**Results:**

Of the 267 studied patients, 14 developed neurological complications (5.2%); seven developed chorea (2.6%), four developed seizures (1.5%), and two developed both seizure and chorea (0.7%). One case developed subarachnoid hemorrhage (SAH). We observed that age (*p* = 0.000), weight (*p* = 0.000), and RACHS score (*p* = 0.006) were associated with the development of neurological complications. Additionally, we observed that “neutrophil to lymphocyte ratio” was not associated with the risk of postsurgical neurological complications.

**Conclusion:**

Younger age, lower weight, and higher RACHS score were associated with neurological complications after operations. Given the importance of postsurgical neurological complications, further investigations should be carried out to cover this issue and discover preventive strategies for such morbidities.

## 1 INTRODUCTION

Over the past years, the survival rate in pediatric heart surgery has increased due to novel surgical techniques. Therefore, postsurgery morbidities such as neurological complications require more attention. Indeed, neurological complications including strokes, seizures, chorea, and impaired motor function are still critical risk factors for morbidity after cardiac surgery.^1^, ^2^, ^3^, ^4^, ^5^ Furthermore, since the central nervous system (CNS) in the infancy and neonatal period is immature, the chance of remaining neurological sequelae such as speech and language disorders and motor deficits increase. On the other hand, it should be noted that neurological deficits could exist simultaneously with cardiac diseases before surgical intervention in pediatric patients.

Several preoperative, intraoperative, postoperative, and prenatal factors affect the neurological complications that appear following cardiac surgery.^6^ Accordingly, some of the reported factors are the following: brain immaturity, hemodynamic instability, anesthetic technique, and intensive care unit (ICU) length of stay.^6^ In this cross‐sectional study, we investigated the incidence of neurological complications in the pediatric cardiac intensive care unit at the children's medical center following cardiac surgery. It also discusses its association with sex, weight, age, and neutrophil to lymphocyte ratio (NLR). We chose NLR, because it has been shown to be prognostic in many acute conditions such as cancer,^7^, ^8^ sepsis,^9^, ^10^, ^11^, ^12^, ^13^ surgery,^14^, ^15^, ^16^ and even Covid‐19.^17^

## 2 MATERIALS AND METHODS

In this cross‐sectional study, carried out in Children's Medical Center, from March 2019 to September 2019, we aimed to investigate the incidence of neurologic complications. Furthermore, we evaluated its association with the NLR.

We included all the patients referred to us with cardiovascular disorders, who underwent either open or closed heart procedures, and were later transferred to the open‐heart intensive care unit. We excluded patients who passed away during their admission for heart procedures or had incomplete medical files.

We included all the patients who had been admitted to the cardiac ICU (complete enumeration) and utilized the medical records of patients who demonstrated acute neurologic disorders from the time of their procedure to discharge from the hospital. We recorded their age, sex, type of congenital heart defect, the presence of seizure, chorea, stroke, their time of operation, risk adjustment for congenital heart surgery (based on the Risk Adjustment for Congenital Heart Surgery [RACHS‐1] risk categories), and NLR in the first and second day after their procedure. We also reviewed their CT scan results and their radiology reports.

We estimated the sample size by considering a 5% margin of error, 0.2 probability of complications (based on the current literature), 95% confidence level, and reached the number of 246 patients. Finally, we included 267 patients in our study. Upon receiving informed consent, we entered the data in SPSS version 28 (IBM Corp. Released 2021. IBM SPSS Statistics for Mac, Version 28.0. IBM Corp), and performed the appropriate two‐sided statistical tests, with *p* = 0.05 considered as the threshold for statistical significance. We used the *χ* ^2^ test or Fisher's exact test for qualitative variables (sex and different age groups), and Independent *t*‐test or nonparametric equivalents for quantitative variables (weight, surgical risk, and ratios). We used Logistic regression (Enter method) to investigate the association between all the variables and neurologic complications. We have mentioned the type and name of the statistical test that was utilized at every stage, along with the relevant values, *p* values, and their significance.

Our study was reviewed and approved by the board of medical ethics at Tehran University of Medical Sciences with the ethics code of “IR.TUMS.CHMC.REC.1398.056.”

## 3 RESULTS

We studied 284 children. 157 (55.3%) were male and 127 (44.7%) were female. The average age of children was 32.014 (standard deviation = 37.6) months. The youngest was 5 days old, the eldest was 17 years old, and the rest were 14 years old or younger. The children weighed an average of 10.932 (8.6) kilograms. We observed that 14 (5.2%) patients developed neurological complications. Seven of whom developed chorea, four developed seizures, and two developed both seizure and chorea simultaneously. In addition, one case of subarachnoid hemorrhage (SAH) was observed. The summary of patients' characteristics is listed in Table 1.

**Table 1:** Summary of the patients' characteristics

Variable	Groups/units	Mean (SD) or number (%)
Age	Months	32.014 (37.622)
Sex	Male	148 (55.3%)
	Female	119 (44.7%)
Weight	Kilograms	10.932 (8.585)
Neurologic event	Chorea	7 (2.6%)
	Seizure	4 (1.5%)
	Chorea and seizure	2 (0.7%)
	Subarachnoid hemorrhage	1 (0.4%)
	No complications	253 (94.8%)
RACHS score		2.313 (0.749)
WBC count first‐day postoperation	Number per milliliters	12,551 (6886)
WBC count second‐day postoperation	Number per milliliters	14,388 (4798)
ANC first‐day postoperation	Number per milliliters	9056 (5924)
ANC second‐day postoperation	Number per milliliters	11,189 (4462)
ALC first‐day postoperation	Number per milliliters	2801 (1850)
ALC second‐day postoperation	Number per milliliters	6950 (964)
NLR first‐day postoperation	N/A (ratio)	4.037 (2.970)
NLR second‐day postoperation	N/A (ratio)	7.795 (5.422)

Abbreviations: ALC, absolute lymphocyte count; ANC, absolute neutrophil count; N/A, not applicable; NLR, neutrophil to lymphocyte ratio.

The presence of neurologic complications did not demonstrate a statistically significant association with the sex of the children (*p* = 0.197). 10 out of 148 males, and 4 out of 119 female children developed neurologic complications.

The average age of the children who developed neurologic complications was 10.4 (30.1), and 34 months (37.9) for those who did not develop neurologic complications. The children who developed neurologic complications were significantly younger (*p* < 0.001). Among the 14 children who showed neurologic complications, 3 were younger than 1 month old, 10 were between 1 month and 1 year old and 1 was older than 5 years old. Categorizing the children by 1 year old cut‐off, 13 of 14 children with neurologic events were younger than 1 year old (*p* < 0.001).

The average weight (kg) of the children without complications was 11.3 (8.6), and 5.4 (5.7) for those who developed complications. The children with neurological complications weighed significantly less than those without complications (*p* < 0.001).

The surgical risk of patients was calculated using the RACHS‐1 scoring system. The children were scored between 1 and 4. Overall, the children had an average of 2.3 (0.7) RACHS‐1 score. On average, those who developed complications had a score of 2.8 (0.7), and those who did not have a complication had an average score of 2.3 (0.7). The children who developed complications had a significantly higher RACHS‐1 score (*p* = 0.006).

The average of White Blood Cell (WBC) counts on Day 1 postoperation was 12,551 (6886), and 14,388 (4798) on Day 2 postoperation (*p* < 0.001). The absolute Neutrophil count per milliliter was 9056 (5924) on Day 1 postoperation and 11,189 (4461) on Day 2 postoperation (*p* < 0.001). The absolute lymphocyte count per milliliter was 2801 (1849) on Day 1 postoperation and 1837 (964) on Day 2 postoperation (*p* < 0.001). NLR was 4.04 (2.97) on Day 1 postoperation and 7.79 (5.4) on Day 2 postoperation (*p* < 0.001). The NLR on the first‐day postoperation among those with complications was less than the children who did not have complications, but this difference was not statistically significant (2.8 vs. 4.2, *p* = 0.099). The NLR on the second‐day postoperation was lower among those with complications (7.2 vs. 7.9); however, this difference was not significant either (*p* = 0.824). RACHS score did not demonstrate a significant association with either NLR of the first day (Kendal's Tau coefficient: −0.053, *p* = 0.275) or that of the second day (Kendal's Tau coefficient: −0.06, *p* = 0.339).

The summary of univariate analyses is listed in Table 2.

**Table 2:** Univariate analysis of the association between neurologic complications and independent variables

Variable	Groups/units	Mean/number	Presence of neurologic complications (number/mean)		*p* Value	Statistical test
			Yes	No		
Sex	Male	148	10	138	0.197	Fisher's Exact
	Female	119	4	115		
Age	Months	32.01	10.4 (30.1)	34 (37.9)	<0.001*	Mann–Whitney U
Age groups	≤1 month	33	3	30	0.001*	Fisher's Exact
	1 month to 1 year	89	10	79		
	1 year to 5 years	86	0	86		
	>5 years	52	1	51		
Age binary groups	≤1 year old	122	13	109	<0.001*	Pearson's *χ* ^2^
	>1 year old	138	1	137		
Weight	Kilograms	11.1	5.4 (5.7)	11.3 (8.6)	<0.001*	Mann–Whitney U
Surgical risk	RACHS score (1–4)	2.313	2.8 (0.7)	2.3 (0.7)	0.006*	Mann–Whitney U
NLR first‐day postoperation		4.037	2.8 (1.6)	4.2 (3)	0.099	Mann–Whitney U
NLR second‐day postoperation		7.795	7.2 (10.2)	7.9 (5.6)	0.824	Mann–Whitney U

*Note*: The *p* values that showed statistical significance are marked with an asterisk and bold font.

Abbreviation: NLR, neutrophil to lymphocyte ratio.

In multivariate analysis, using enter method of logistic regression, only weight demonstrated a significant association with adverse neurologic events (*p* = 0.011).

## 4 DISCUSSION

This study evaluated the incidence of neurological complications in 267 children who underwent cardiac surgery due to a congenital heart disease. The range of age in children following surgery was from 5 days to 17 years. According to the results, 14 (5.2%) children developed neurological complications; four patients developed seizures (1.5%), seven developed chorea (2.6%), and two developed both seizure and chorea (0.7%). Moreover, one patient with SAH was detected. Of note, lower weight, age, and RACHS score were evaluated as risk factors for developing neurological complications.

Similarly, Arslanoğlu et al. reported in a recent study that in a population of 3849 children, 162 (4.2%) showed neurological complications within an early period following cardiac surgery. Furthermore, 69 of those 162 patients (42.6%) presented with seizures.^18^ Of note, the authors suggested that developing neurological complications soon after surgery was significantly associated with increasing the risk of mortality. They, like us, believed the importance of neurological complications in the prognosis of those children, and that studies in this important area are lacking. While they looked for factors affecting mortality, we looked for factors in occurrence of those neurological events, with focus on neutrophile‐to‐lymphocyte ratio.^18^ Likewise, Ghosh et al. studied the seizure risk factors in 247 infants and neonates <3 months with congenital heart disease in the United States in 2020. According to the results, 2.4% developed seizures in the early preoperative period, 1.6% early postoperative period, and 5.3% in the late postoperative.^19^ Furthermore, Jafri et al. reported that out of 2000 pediatric patients 35 presented with acute neurological complaints (1.75%). Notably, 80% of those with neurological symptoms developed seizures.^20^ However, Desnous et al. studying 128 children with complex congenital heart disease, observed a higher incidence of the perioperative clinical seizure.^21^ In this study, 10 patients (comprising a rate of 7.8%) developed perioperative seizures. However, the smaller study population and lower mean age of patients might explain the disparity between results.

Postpump chorea (PPC) is known as choreoathetoid movements developing within 2 weeks following cardiopulmonary bypass surgery in children.^22^ The incidence of PPC in our study population was 3.9%. Similarly, Medlock et al. observed 668 pediatric patients who underwent cardiac surgery over a 10‐year period; according to their findings, 8 patients (1.2%) developed PPC.^23^ Notably, by comparing patients with PPC with controls, a strong relationship was found between developing PPC, and deep hypothermia and circulatory arrest. However, future studies should provide more detailed information regarding the incidence and etiology of developing chorea following pediatric surgery.

Based on our results, lower NLR was associated with a higher risk of developing neurological symptoms. In the first day the difference among the two groups were more pronounced that second day; however, both associations were not significant (*p* = 0.099 & *p* = 0.824). This finding in our study contradicts some of the other studies to date.^7^, ^9^, ^10^, ^17^, ^24^ Furthermore, we did not discover any relationship between the sex of the patients and the presence of neurological symptoms. Notably, we observed that younger children were more susceptible to present with neurological complications. In addition, lower weight was significantly associated with developing neurological complications. Consistently, Arslanoğlu et al. reported a lower mean age and weight among children with intracranial bleeding. However, according to their findings, lower mean age and weight of the patients were not associated with the increased mortality risk.^18^ A recent retrospective cohort study aimed to investigate the effect of the NLR on the outcome of open‐heart surgery with cardiopulmonary bypass (CPB) in children.^25^ According to the findings, higher postoperative levels of NLR predicted longer postoperative cardiac ICU stay and delayed extubating time. Moreover, another recent study on 424 infants reached consistent results; accordingly, increased postoperative NLR was associated with increased mechanical ventilation time, longer ICU and hospital stay.^24^ Overall, it was observed in other studies that higher levels of postoperative NLR seems to be an important predictor for poor outcomes following pediatric cardiac surgery, independent of other variants.^24^, ^25^

## 5 STRENGTHS AND LIMITATIONS

While other studies have been working on what happens after the fact, we have tried to look for factors in predicting those neurological complications. Also, children's medical center is a referral center, and while the number of cases in our center are more than other centers, the results of our study might not be representative of the entire population. The patients who are referred to us, are already some of the highest‐risk patients. It is therefore plausible to estimate that in a lower risk population, the outcome could be different, considering the *p* = 0.09 of NLR in the first‐day postoperative. Also, another limitation of our study was that EEG abnormality in the absence of clinical seizures could not be detected.

## 6 CONCLUDING REMARKS

Neurological complications following congenital heart surgery are still an important cause of postoperative mortality and morbidity in the pediatric population. Furthermore, neurological complications in pediatric patients tend to last longer than in adults. In our study, we evaluated the incidence of postoperative neurological complications. We also addressed the hypothesis that postoperative NLR affects the incidence of neurological complications and poor outcomes following pediatric heart surgery. However, other risk factors associated with the increased incidence of neurological complications await more investigation; therefore, future studies should unlock new strategies to prevent and/or halt the progression of postoperative neurological morbidities.

## AUTHOR CONTRIBUTIONS

**Alireza Abdshah**: Formal analysis; investigation; writing – original draft; writing – review and editing. **Mohammadreza Mirzaaghayan**: Conceptualization; data curation; investigation; project administration; resources; supervision. **Morteza Heidari**: Data curation; investigation; project administration; resources. **Sara Memarian**: Data curation; investigation; writing – original draft. **Mobina Amanollahi**: Data curation; writing – original draft; writing – review and editing. **Azadeh Nazeri**: Data curation; investigation; writing – original draft. **Behdad Gharib**: Conceptualization; data curation; project administration; supervision.

## CONFLICT OF INTEREST

The authors declare no conflicts of interest.

## ETHICS STATEMENT

The study was reviewed and approved by the board of medical ethics at Tehran University of Medical Sciences, with the code: “IR.TUMS.CHMC.REC.1398.056.”

## TRANSPARENCY STATEMENT

The lead author Behdad Gharib affirms that this manuscript is an honest, accurate, and transparent account of the study being reported; that no important aspects of the study have been omitted; and that any discrepancies from the study as planned (and, if relevant, registered) have been explained.

## Data Availability

Data can be made available upon request.
